# Alternative *UNC13D* Promoter Encodes a Functional Munc13-4 Isoform Predominantly Expressed in Lymphocytes and Platelets

**DOI:** 10.3389/fimmu.2020.01154

**Published:** 2020-06-09

**Authors:** Donatella Galgano, Tayebeh Soheili, Matthias Voss, Lamberto Torralba-Raga, Bianca Tesi, Frank Cichocki, Isabelle Andre, Jens Rettig, Marina Cavazzana, Yenan Bryceson

**Affiliations:** ^1^Department of Medicine, Center for Hematology and Regenerative Medicine, Karolinska Institutet, Stockholm, Sweden; ^2^Human Lymphohematopoiesis Laboratory, INSERM UMR 1163, IMAGINE Institute, Paris, France; ^3^Department of Clinical Genetics, Karolinska University Hospital, Stockholm, Sweden; ^4^Department of Molecular Medicine and Surgery, Center for Molecular Medicine, Karolinska Institutet, Stockholm, Sweden; ^5^Division of Hematology, Oncology and Transplantation, University of Minnesota, Minneapolis, MN, United States; ^6^Paris Descartes-Sorbonne Paris Cité University, Imagine Institute, Paris, France; ^7^Cellular Neurophysiology Laboratory, Center for Integrative Physiology and Molecular Medicine, Saarland University, Homburg, Germany; ^8^Biotherapy Department, Hôpital Necker-Enfants malades, Assistance Publique-Hôpitaux de Paris, Paris, France; ^9^Biotherapy Clinical Investigation Center, Groupe Hospitalier Universitaire Ouest, Assistance Publique-Hôpitaux de Paris, INSERM, Paris, France; ^10^Broegelmann Research Laboratory, Department of Clinical Sciences, University of Bergen, Bergen, Norway

**Keywords:** *UNC13D*, primary immunodeficiency, familial hemophagocytic lymphohistiocytosis type 3, intronic mutation, alternative intronic promoter/isoform, lymphocyte cytotoxicity

## Abstract

Autosomal recessive mutations in genes required for cytotoxicity are causative of a life-threatening, early-onset hyperinflammatory syndrome termed familial hemophagocytic lymphohistiocytosis (FHL). Mutations in *UNC13D* cause FHL type 3. *UNC13D* encodes Munc13-4, a member of the Unc13 protein family which control SNARE complex formation and vesicle fusion. We have previously identified FHL3-associated mutations in the first intron of *UNC13D* which control transcription from an alternative transcriptional start site. Using isoform specific antibodies, we demonstrate that this alternative Munc13-4 isoform with a unique N-terminus is preferentially expressed in human lymphocytes and platelets, as compared to the conventional isoform that was mostly expressed in monocytes and neutrophils. The distinct N-terminal of the two isoforms did not impact on Munc13-4 localization or trafficking to the immunological synapse of cytotoxic T cells. Moreover, ectopic expression of both isoforms efficiently restored exocytosis by FHL3 patient-derived Munc13-4 deficient T cells. Thus, we demonstrate that the conventional and alternative Munc13-4 isoforms have different expression pattern in hematopoietic cell subsets, but display similar localization and contribution to T cell exocytosis. The use of an alternative transcriptional starting site (TSS) in lymphocytes and platelets could be selected for increasing the overall levels of Munc13-4 expression for efficient secretory granule release.

## Introduction

Cytotoxic T lymphocytes and natural killer (NK) cells constitute the major lymphocyte subpopulations capable of rapid target cell killing. They contain a specialized secretory lysosomal compartments, termed cytotoxic granules (CG) (1). Upon target cell recognition, an immunological synapse (IS) is formed at the interface between the effector and target cell to which CGs are trafficked. Granule contents, including proteins like perforin and granzymes, are released into the IS cleft by exocytosis, triggering target cell death (2). Autosomal recessive mutations in genes required for CG-dependent lymphocyte cytotoxicity have been associated with a life-threatening, early-onset, hyperinflammatory syndrome termed familial hemophagocytic lymphohistiocytosis (FHL) (3, 4). FHL is a rare primary immunodeficiency syndrome that typically presents with unremitting fever, hepatosplenomegaly, hyperferritinemia, cytopenia, hemophagocytosis and sometimes central nervous system involvement. The hyperinflammation is treated with radical immunosuppression, but to date hematopoietic stem cell transplantation (HSCT) represents the only cure for FHL (5). Besides *PRF1*, encoding perforin and associated with FHL2, three genes encoding proteins involved in CG exocytosis have been associated with FHL. *UNC13D, STX11*, and *STXBP2*, encode Munc13-4, Syntaxin-11 (Stx11), and Munc18-2, respectively. Autosomal recessive mutations in either of these three genes cause FHL3, FHL4, or FHL5 (3, 4). Functional analyses of cytotoxic lymphocytes from these patients have provided insights to different molecular steps of CG exocytosis.

Munc13-4 belongs to a family of four homologous proteins. In neuronal cells, Munc13-1, -2, and -3 bridge the plasma membrane and vesicle, playing an essential role in synaptic vesicle priming and exocytosis. Their function can be regulated through alternative splicing of their N-termini. Distinct neuronal Munc13 N-termini differentially modulate Munc13 localization to the plasma membrane and thereby exocytic function (6, 7). Munc13-4 is more widely expressed, with high expression in hematopoietic cells. In FHL3 patients' T and NK cells, CG dock normally at the IS membrane but cannot fuse, thereby implicating Munc13-4 in the final step of CG-to-plasma membrane fusion (8, 9). How Munc13-4 binds the plasma membrane and is activated is not known. Intriguingly, mutations in an evolutionary conserved region of *UNC13D* intron 1 have been identified as a frequent cause of FHL3 (10–13). This sequence represents an overall enhancer and also controls expression of an alternative Munc13-4 isoform with a unique N-terminus (10, 14). In lymphocytes, alternative *UNC13D* transcript expression was abrogated in a patient homozygous for the *UNC13D* c.118-308C>T intronic mutation, with conventional *UNC13D* transcript levels also being severely reduced (14). Lymphocyte exocytosis is abrogated in patients carrying the *UNC13D* c.118-308C>T intronic mutation, indicating that any residual transcription of the conventional *UNC13D* isoform could not support cytotoxic lymphocyte exocytosis (14). Nonetheless, conventional *UNC13D* transcripts were abundantly expressed in other immune cell types of these patients. Of note, an additional intron 1 *UNC13D* c.117+143A>G variant has been identified in a patient diagnosed with recurrent macrophage activation syndrome (MAS) and systemic juvenile idiopathic arthritis (15), providing further evidence that *UNC13D* intron 1 is important for transcriptional regulation of the gene. No disease causing *UNC13D* mutations have been reported in the first coding exon of the conventional *UNC13D* transcript, suggesting that the conventional Munc13-4 isoform might be redundant. In terms of the alternative Munc13-4 isoform originating from *UNC13D* intron 1, its expression and potential capacity to mediate exocytosis has not been determined. We hypothesized that the distinct N-termini of Munc13-4 isoforms may differentially regulate their exocytic function, potentially correlating with different requirements for vesicle trafficking and exocytosis in distinct immune cells.

To gain insights into the function of the two major human Munc13-4 isoforms, we compared their expression in different hematopoietic cell types, examined their trafficking and quantified their contribution to exocytosis in T cells. We establish that the conventional and alternative Munc13-4 isoforms have different expression pattern in hematopoietic cells, yet display a similar ability to restore exocytosis of Munc13-4-deficient T cells from FHL3 patients.

## Materials and Methods

### Cells

All human material was collected with written informed consent in accordance with the Declaration of Helsinki II. This study was approved by the Regional Ethics Review Board in Stockholm (2006/229-31/3; 2013/1273-31/4) and Ethics Commission of the Saarland University Medical Center (2015/83/15; 2015/84/15). Human peripheral blood mononuclear cells (PBMCs) were isolated from healthy volunteers as well as patients by Ficoll density gradient centrifugation (Lymphoprep; Axis-Shield). The human embryonic kidney epithelial HEK-293T cell line was used for transient transfections (periodically tested for *Mycoplasma* contamination) and maintained in Dulbecco's modified Eagle's medium (DMEM; ThermoFisher Scientific) supplemented with 10% fetal bovine serum (FBS; Sigma Aldrich).

For protein expression analyses, platelets and neutrophils were purified from plasma fraction and granulocyte-erythrocyte fraction, respectively, following Ficoll density gradient centrifugation. For platelet isolation, 10 mL platelet-rich plasma (PRP) was gently mixed at 1:1 ratio (v/v) with HEP buffer (140 mM NaCl, 2.7 mM KCl, 3.8 mM HEPES, 5 mM EGTA, pH 7.4.) and subsequently centrifuged at 100 *g* for 15 min at room temperature without brake to pellet contaminating red and white blood cells. The supernatant was centrifuged at 800 *g* for 15 min at room temperature without brake to enrich the platelet fraction. The platelet pellet was gently washed twice with platelet wash buffer [10 mM sodium citrate, 150 mM NaCl, 1 mM EDTA, 1% (w/v) dextrose, pH 7.4.]. Finally, the pellet was slowly resuspended in Tyrode's buffer (134 mM NaCl, 12 mM NaHCO_3_, 2.9 mM KCl, 0.34 mM Na_2_HPO_4_, 1 mM MgCl_2_, 10 mM HEPES, pH 7.4). For neutrophil isolation, the collected erythrocyte-granulocyte fraction was pelleted and washed for 25 s in 12 ml ice-cold sterile distilled water to remove contaminating erythrocytes by hypotonic lysis. Hypotonic lysis was terminated by adding 4 ml of 0.6 M KCl (final 0.15 M KCl) to restore isotonicity. The mixture was centrifuged at 500 *g* for 5 min at 4°C. This step was repeated 2–3 times until no more red blood cells were visible. Neutrophils were then resuspended in phosphate buffered saline (PBS). Enriched platelets and neutrophils were then counted, 1 × 10^5^ cells were stained for purity assessment by flow cytometry and the remaining cells were centrifuged at 500 *g* for 5 min prior to cell lysis. For cell sorting, PBMCs from healthy donors were stained with fluorochrome-conjugated antibodies and incubated for 30 min on ice and sorted with a FACSAria flow cytometer (BD Biosciences). Monocytes (FSC^high^SSC^high^CD14^+^), B cells (CD3^−^CD56^−^CD19^+^ lymphocytes), naïve CD4^+^ T cells (CD3^+^CD4^+^CD28^+^CD45RA^+^CCR7^+^CD57^−^ lymphocytes), memory CD4^+^ T cells (CD3^+^CD4^+^CD28^+^CD45RA^−^CCR7^+/−^CD57^−^ lymphocytes), naïve CD8^+^ T cells (CD3^+^CD8^+^CD28^+^CD45RA^+^CCR7^+^CD57^−^ lymphocytes), memory CD8^+^ T cells (CD3^+^CD8^+^CD28^+^CD45RA^−^CCR7^+/−^CD57^−^ lymphocytes), effector CD8^+^ T cells (CD3^+^CD8^+^CD28^−^ CD45RA^+^CD57^+^ lymphocytes) and NK cells (CD3^−^CD56^+^ lymphocytes) were sorted. The cell subsets were sorted to more than 98% purity, collected in complete medium, and washed twice in ice-cold PBS prior to cell lysis.

For microscopy experiments, primary cytotoxic CD8^+^ T cells were isolated by negative magnetic selection (CD8^+^ negative isolation kit; Invitrogen-Thermo Fisher Scientific) from the peripheral blood of healthy volunteers and cultured in AIM V medium (Invitrogen-Thermo Fisher Scientific) supplemented with 10% FBS. For cytotoxic T lymphocyte (CTL) generation, freshly isolated CD8^+^ T cells were stimulated for 2 days at a density of 1.5 × 10^6^ cells/ml in AIM V medium supplemented with 10% FBS and anti-CD3/CD28 coated-magnetic beads (Dynabeads Human T-activator, Invitrogen-Thermo Fisher Scientific) according to the manufacturer's instructions.

### Plasmids, Antibodies and Reagents

The coding sequence corresponding to the conventional *UNC13D* transcript was PCR-amplified from a cDNA clone (IMAGE clone 5951944, Source Bioscience) using primers 5′-ATGCGCTAGCACCATGGCGACACTCCTCTCC-CATCCG-3′ and 5′-GCATCTCGAGCTACGGTGCCGGCCGCAAGGCATG-3′ and cloned into the pMax vector (Lonza). From this construct a fragment was excised with *Nhe*I/*Sda*I and replaced with a synthetic gBlock fragment (IDT) corresponding to the alternative exon 1 sequence in EST clone CR983520 to generate an expression construct for the alternative exon 1 transcript. In addition, chimeric C-terminally mCherry-tagged (linker: GGSGGSGGS) variants of both *UNC13D* constructs were cloned into a modified pMax vector. All plasmids were verified by bidirectional sequencing. The coding sequence corresponding to human *RAB11A* was PCR-amplified from human cDNA using 5'-TATATGAATTCTATGGGCACCCGCGACGAC-3' and 5'-TATATAGGATCCTTAGATGTTCTGACAGCACTG-3' primers with *Eco*R1/*Bam*H1 sites and cloned into the pGFP-C1 plasmid. Granzyme B-mTFP plasmid has been previously described (16).

For generation of lentiviral expression constructs, the cDNA of each hMunc13-4 isoform was subcloned into a self-inactivating (SIN) pCCLΔU3 lentiviral plasmid in frame with a C-terminal GFP encoding sequence, downstream of an elongation factor 1 alpha (EF1α) promoter. The hMunc13-4 expressing pCCLΔU3 plasmid was used to produce lentiviral particles pseudotyped with VGV-G envelope (17). The lentiviral vector titer was assessed by droplet digital PCR on isolated genomic DNA of HCT116 cell-line, infected with a serial dilution of the lentiviral suspension, using TaqMan probes designed to detect a lentiviral sequence (Psi) and human Albumin sequence.

For purity assessment of platelet and neutrophil fraction, anti CD42a-FITC (clone HIPI, BioLegend), anti-CD61 (Clone VI-PL2; BioLegend), anti-CD16-PE (clone 3G8; Becton- Dickinson BD), anti-CD15-BUV395 (clone W6D3; BD), anti CD3-Brilliant Violet785 (clone OKT3; Biolegend), anti-CD56-PE Cy7 (clone NCAM16.2; BD), anti-CD19-APC (clone SJ25C1; BD) and anti-CD14-APCCy7 (clone MϕP9, BD) were used. For FACS sorting, anti-CD3ε-FITC (clone SK7; BD), anti-CD4-Qdot605 (clone S3.5; Invitrogen-Thermo Fischer Scientific) anti-CD8-Brilliant Violet 711 (clone SK-1; BioLegend), anti-CD14-APCCy7, (clone MϕP9, BD), anti-CD19-APC, (clone SJ25C1; BD), anti-CD28-PE-CF594 (clone NK-1; Beckman coulter), anti-CD45RA-Brilliant Violet650 (clone H100; BioLegend), anti-CD56-PE Cy7 (clone NCAM16.2; BD), anti-CCR7-Pacific Blue (clone G043H7, BioLegend) and LIVE/DEAD Fixable Aqua Dead Cell Stain (Thermo Fischer Scientific) were used. For Western blotting, monoclonal rabbit anti-conventional Munc13-4 (MAB89662) and anti-alternative Munc13-4 (MAB8966) antibodies have been made commercially available (R&D Systems). The polyclonal rabbit anti-Munc13-4 antibody (16905-1-AP, ProteinTech Group), the monoclonal anti-β**-**actin HRP (clone AC-15, Merck-Sigma Aldrich), and polyclonal rabbit anti-GAPDH (MAB374; Merck-Millipore) were also used. For total internal reflection fluorescence (TIRF) microscopy, monoclonal anti-CD3 (clone B-B11; Diaclone) was used for cell stimulation.

### *UNC13D* Transcription Analysis

For FANTOM5 analysis, data were downloaded from http://fantom.gsc.riken.jp/5/data/ (on July 2nd, 2019). The data set consisted of 5'cap mRNA transcript peaks for human samples in the form of relative log expression normalized data. Data from biological replicates were averaged. Three donors from each subset were analyzed, unless otherwise indicated. Data from dermal fibroblast (*n* = 6), CD34^+^ progenitors (*n* = 2), CD34^+^ stem cells from adult bone marrow (*n* = 1), CD34^+^ cells differentiated to erythrocyte lineage, total PBMCs, isolated neutrophils, CD19^+^ B cells, classical CD14^+^CD16^−^ monocytes, intermediate CD14^+^CD16^+^ monocytes, non-classical CD14^−^CD16^+^ monocytes, CD4^+^ T cells, CD8^+^ T cells, NK cells, dendritic cells derived from immature monocytes, plasmacytoid dendritic cells, monocyte-derived macrophages, granulocyte/macrophage progenitor, and the leukemia chronic megakaryoblastic cell line MEG-01 were examined. For sequence comparison, *UNC13D* gene sequences for *Homo sapiens* (NG_007266.1), *Macaca mulatta* (NC_027908.1), *Mus musculus* (NC00077.6), *Rattus norvegicus* (NC_005109.4), and *Canis lupus familiaris* (NC006591.3) were downloaded from NCBI-Ref seq (https://www.ncbi.nlm.nih.gov/refseq/). The alignment was performed using Clustal W Genome Net at https://www.genome.jp/tools-bin/clustalw.

### Cell Transfection and Nucleofection

For transient transfection of HEK-293T cells, cells were cultured overnight in a poly-L-lysine coated 6-cm tissue culture petri dish and then transiently transfected with plasmid DNA encoding human Munc13-4 isoforms or GFP using Lipofectamine 2000 and OptiMEM medium (Thermo Fisher Scientific) according to the manufacturer's instructions. Medium was replaced 24 h after transfection and 48 h after transfection cells were harvested in ice-cold PBS and subsequently lysed. After 2 days of bead stimulation, 5 × 10^6^ CTLs were transiently transfected with 1.5 μg of plasmid DNA using Amaxa nucleofection technology (4D Amaxa Human T Nucleofector Kit, Lonza). Cells were imaged 18 h after transfection.

### Western Blotting

For assessment of Munc13-4 expression, 0.5 × 10^6^ transfected HEK-293T cells, primary neutrophils, platelets, or sorted cell subsets were lysed in 1% Triton X-100 (Sigma Aldrich), 1% NP-40 (IGEPAL CA-630, Sigma Aldrich) in 50 mM Tris-HCl, 150 mM NaCl 2 mM EDTA pH 7.6 and 1X Halt Protease Inhibitor Cocktail (Thermo Fischer Scientific), 1 mM Na_3_VO_4_ (Sigma Aldrich), 1 mM PMSF for 1 h on ice. Lysates were cleared by centrifugation at 16,000 *g* for 20 min and boiled 10 min in 1 × NuPage LDS sample buffer (Thermo Fisher Scientific) and Dithiothreitol (Thermo Fisher Scientific). The proteins were resolved by SDS-PAGE gel electrophoresis (Invitrogen-Thermo Fisher Scientific) and transferred onto nitrocellulose membranes using fast semi-dry transfer method (iBlot 2 Dry Blotting System; Invitrogen-Thermo Fisher Scientific). Membranes were blocked in 5% bovine serum albumin (BSA, Sigma Aldrich) in PBS for 1 h at RT and probed with the indicated antibodies overnight followed by horseradish peroxidase (HRP)-conjugated secondary antibody (Thermo Fisher Scientific). Labeled antibodies were detected using the enhanced chemiluminescence (ECL) kit (SuperSignal West Dura Extended Duration Substrate, Thermo Fischer Scientific). Blots were scanned using Licor Odyssey (Li-COR) and quantified using Image Studio Lite software (Li-COR).

### Confocal Microscopy

For confocal microscopy, CTLs were electroporated with an EGFP-Rab11a construct as a RE marker or GranzymeB-TFP as a marker of CGs, as well as conventional or alternative Munc13-4-mCherry constructs. Eighteen hours post-transfection, CTLs were plated onto poly-L-ornithine hydrobromide (Sigma Aldrich) coated 12 mm round cover slips (0.17 mm thickness, Carl Zeiss). The cells were allowed to adhere for 15 min and then fixed by incubation in 4% paraformaldehyde (Sigma Aldrich) in PBS for 20 min at room temperature (RT). The slides were washed in PBS and mounted in mounting medium (ProLong Antifade Kit, P7481, Thermo Fisher Scientific). For staining of mitochondria, 0.2 × 10^6^ cells were stained with 250 nM MitoTracker Deep red (Invitrogen-Thermo Fisher Scientific) in pre-warmed PBS at 37°C for 30 min. Cells were then washed, and resuspended in PBS, and plated on coverslips. For stimulation, 0.2 × 10^6^ CTL were incubated in pre-warmed media alone or stimulated with 100 ng/mL phorbol 12-myristate 13-acetate (PMA; Calbiochem) and 1 μM ionomycin (Sigma-Aldrich) at 37°C for 20 min prior to adhesion.

Confocal microscopy was performed with a Zeiss LSM 780 (Zeiss) microscope with a 63 × objective. Images were acquired with pinholes opened to obtain 0.8-mm-thick sections. Detectors were set to detect an optimal signal below the saturation limits. Image sets to be compared were acquired using the same acquisition settings. Images were acquired using ZEN software (Zeiss). Images were processed using Fiji Image J v2.0 (NIH, https://imagej.net/Fiji/Downloads). Colocalization analyses to obtain a Manders' overlap coefficient were performed using the JACoP plugin.

### Total Internal Reflection Fluorescence Microscopy

For TIRF microscopy of Munc13-4 isoform trafficking to the IS and association with CGs, CTLs were electroporated with a Granzyme-mTFP construct as cytotoxic granule (CG) marker as well as conventional or alternative Munc13-4-mCherry constructs. Eighteen hours post-transfection, CTLs (0.2–0.3 × 10^6^ cells) were washed and resuspended in 30 μl AIM V medium and allowed to settle in an isotonic Ca^2+^-free modified Ringer's solution (155 mM NaCl, 4.5 mM KCl, 5 mM Hepes, 2 mM MgCl_2_, and 10 mM glucose; 300–310 osM) on coverslips, precoated with poly-L-lysine for 30 min followed by 2 h of incubation on anti-CD3 (30 μg/mL) at 37°C. Cells were then perfused with modified Ringer's solution supplemented with 10 mM Ca^2+^ to optimize Ca^2+^-influx and induce CG fusion. Cells were imaged with a TIRF microscope for 10 min at room temperature (RT = 22 ± 2°C) alternating with lasers at either 561 or 488 nm. The acquisition frequency was 10 Hz, and the exposure time was 100 milliseconds. A TIRFsetup from Visitron Systems GmbH was based on an IX83 microscope system (Olympus) equipped with an autofocus module, a UAPON 100 × OTIRF standard oil immersion objective with NA 1.49, a 488 nm 100 mW laser and a solid-state laser 100 mW emitting at 561 nm, in addition to a iLAS2 illumination control system (Roper Scientific SAS), an evolve- EM 515 camera (Photometrics) and a filter cube containing FF444/520/590/Di01 dichroic and FF01-465/537/623 emission filters (Semrock). The setup was controlled by Visiview software (Version:4.0.0.11, Visitron GmbH). Image sets to be compared were acquired using the same acquisition settings. Images were processed using Fiji Image J v2.0 (NIH). To measure the number of cells harboring conventional or alternative Munc13-4 at the IS, images were acquired after 10 min of stimulation on anti-CD3 coated coverslips upon live imaging. For each image, the number of mCherry^+^ cells and the cells showing Munc13-4 signal in the TIRF plane were calculated using the Cell counter plugin. The data are presented as the percentage of cells harboring Munc13-4/total mCherry^+^ cells detected in the TIRF plane.

### Primary T Cell Transduction

Cryopreserved PBMCs from four unrelated FHL3 patients (Table S1) and healthy donors were cultured in Panserin medium (PAN Biotech; Aidenbach, Germany) supplemented with 5% human AB serum (Dutscher), 100 IU/ml human pro-interleukin-2 (pro-IL2; Novartis) and 1% penicillin- streptomycin at a density of 1.5 × 10^6^ cells/ml. Cells were stimulated with anti CD3-CD28 coated-magnetic beads (Dynabeads Human T-activator) for 48 h in 48-well plates coated with 250 μl (25 μg/ml) RetroNectin reagent (CH296, Takara). Concentrated vector was added at 100 multiplicity of infection (MOI) and incubated overnight at 37°C with the cells at a density of 2 × 10^6^ cells/ml in presence of 0.1 μg/ml LentiBoost (SIRION Biotech). The cells were then washed and seeded into culture plates in the presence of 100 IU/ml pro-IL2 for 5 days prior to functional analyses.

### Flow Cytometric Analysis of Degranulation

Five days after transduction, 0.1 × 10^6^ activated T-cells were incubated with culture medium alone or culture medium supplemented with 3, 10, or 30 μg/ml of the anti-human CD3 antibody (clone OKT3 Miltenyi Biotec) in the presence of PE-conjugated anti-CD107a/b (clone H4A3 and H4B4, BD Biosciences). After 4 h of incubation at 37°C, the cells were stained on ice with the fluorochrome-conjugated antibodies anti-CD3-APC (clone BW264/56, Miltenyi Biotec) and anti-CD8-FITC (clone BW135/80, Miltenyi Biotec), and the cell viability dye 7-aminoactinomycin D (7-AAD) (BD Biosciences) for 30 min and analyzed by flow cytometry (MACS Quant Analyzer, Miltenyi Biotech). The frequency of CD107a/b^+^ cells, as well as CD107 MFI, were determined among total live CD3^+^CD8^+^ GFP^+^ T cells with FlowJo software (v.10; Tree Star Inc).

### Evolutionary Conservation of *UNC13D* Regions

The position in *UNC13D* gene of the exon 1 encoding for the N-terminal of the conventional Munc13-4 isoform, of the intron 1 encoding for the N-terminal of the alternative Munc13-4 isoform and of the exons encoding for the common Munc13-4 domains such as C2B (exons 6–10), MHD1 (exons 20–22), MHD1 (exons 25–28) and C2C (18–20) were mapped using UCSC Genome Browser (University of California Santa Cruz; Human Genome Reference Consortium h37/hg19). The conservation score for each mapped region was calculated using the PhastCons Comparative genomic tool from UCSC (21). For C2B, MHD1, MHD2, and C2C domains, the final score of each domain was calculated as mean of the conservation of each exon computed to the length of the exons.

### Statistical Analysis

Mean values, standard deviation and *p*-values (paired parametric *t*-test, RM-ANOVA, or two-sided Mann-Whitney test) were calculated using GraphPad Prism software (GraphPad Prism Inc.). The threshold for statistical significance was set to *p* ≤ 0.05.

## Results

### Differential Transcription of *UNC13D* Isoforms in Hematopoietic Cells

**Figure 1:**
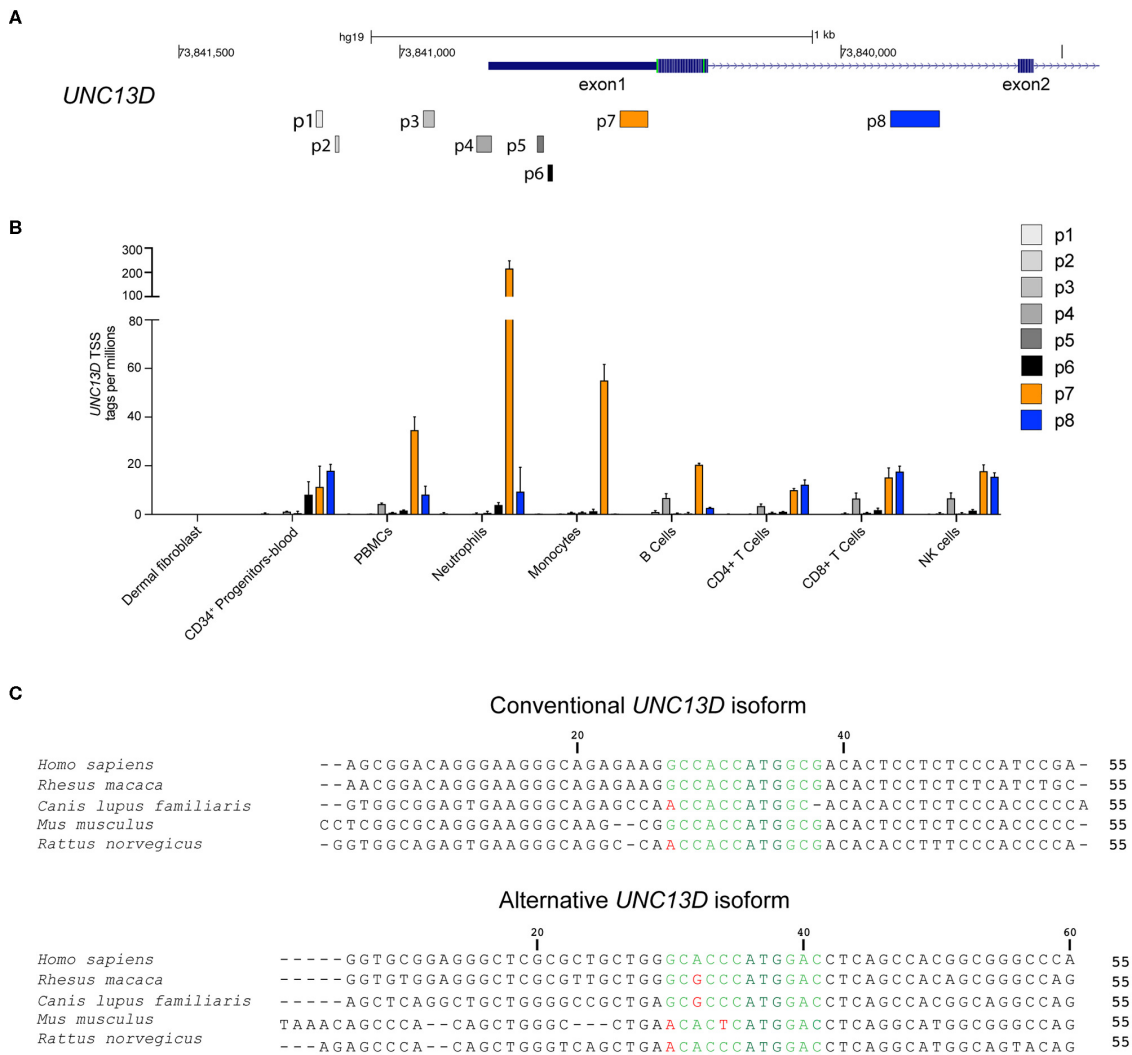
Transcription and Kozak sequence conservation of conventional and alternative *UNC13D* transcripts. **(A)** Localization of *UNC13D* TSS on human chromosome 17 according to FANTOM5 cap analysis gene expression. p7 and p8 represent the conventional and alternative *UNC13D* TSSs. **(B)** The histogram depicts transcription of different *UNC13D* TSS from the FANTOM5 CAGE data of primary fibroblasts as well as total and sorted peripheral blood subsets. **(C)** Alignment of conventional and alternative *UNC13D* Kozak sequences from different mammalian species. The Kozak sequences includes bases flanking the ATG start codon at positions −6/+5. The ATG start codon is colored dark green, conserved and non-conserved residues are in light green and red, respectively.

We have previously presented evidence for differential monocyte and lymphocyte regulation of conventional and alternative *UNC13D* transcripts emanating from distinct transcriptional start sites (TSS) (14). To examine TSS usage across the *UNC13D* locus in different hematopoietic cell subpopulations, we compared the expression profile of seven *UNC13D* transcripts in the FANTOM5 consortium cap analysis of gene expression (CAGE) database (22) (Figure 1A). For comparison, data from fibroblasts was also included. CAGE profiling revealed that the conventional (p7) and alternative (p8) *UNC13D* isoforms are the predominant transcripts in hematopoietic cells. Furthermore, the conventional isoform was the dominant transcript in blood-derived neutrophils, monocytes and B cells, while T cells and NK cells transcribed similar levels of conventional and alternative transcripts (Figure 1B). Blood-derived CD34^+^ progenitor cells also transcribed conventional and alternative *UNC13D* transcripts at a similar level, while erythroid lineage differentiated CD34^+^ cells did not transcribe *UNC13D*. Reflecting transcription of *UNC13D* in peripheral myeloid cells, granulocyte-macrophage progenitors, monocyte-derived macrophages and monocyte-derived dendritic cells preferentially transcribed conventional *UNC13D* (Figure S1). Unlike other myeloid cells, the MEG-01 cell line, a megakaryocyte/platelet-related cell line, and plasmacytoid dendritic cells did not preferentially transcribe the conventional isoform (Figure S1). *UNC13D* transcripts were not detected in dermal fibroblasts (Figure 1B), although analyses of mouse *Unc13d*^−/−^ fibroblasts have indicated a role for Munc13-4 in autophagy (23). Having confirmed differential conventional and alternative *UNC13D* transcription in hematopoietic cells, we determined that both transcripts contain Kozak sequences conserved throughout mammalian evolution (Figure 1C), potentially facilitating translation of the either transcript. Together, these data reveal differential regulation of *UNC13D* TSSs during hematopoietic cell differentiation.

### Differential Expression of Munc13-4 Isoforms in Hematopoietic Cells

**Figure 2:**
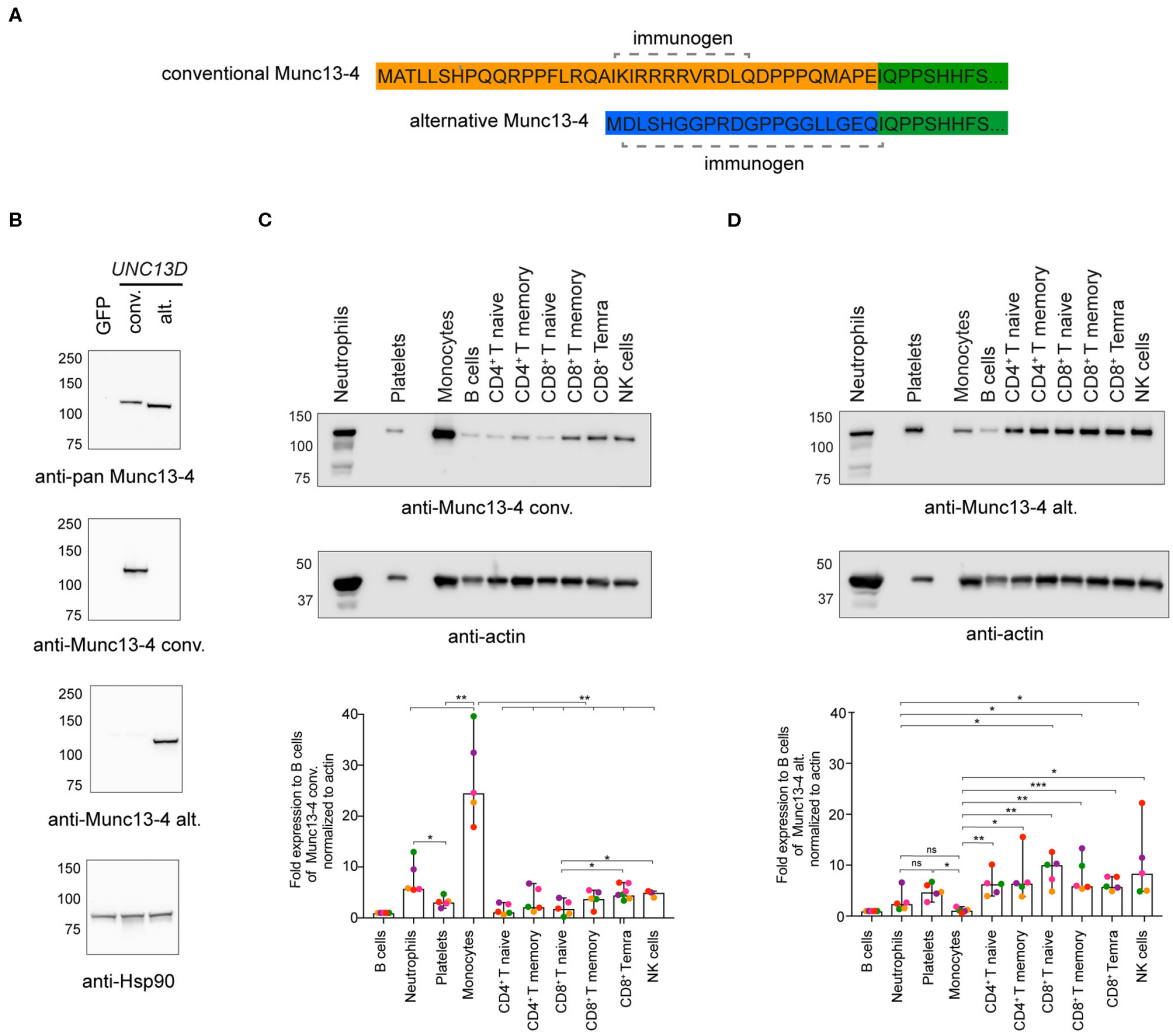
Munc13-4 isoform expression in different human hematopoietic cells. **(A)** Recombinant monoclonal rabbit IgG antibodies were generated against peptides unique to the N-termini of human conventional (conv) or alternative (alt) Munc13-4 isoforms. **(B)** Western blot analysis of whole cell lysates from HEK-293T cells expressing human conventional or alternative Munc13-4. HEK-293T cells, which do not endogenously express Munc13-4, were transiently transfected with plasmids encoding GFP, conventional Munc13-4, or alternative Munc13-4, as indicated. Munc13-4 isoform over-expression was detected using an anti-pan-Munc13-4 antibody, which recognizes a C-terminal epitope shared by both alternative and conventional Munc13-4, or antibodies specific to either isoform. Blotting for Hsp90 served as a loading control. **(C,D)** Western blot analysis of conventional **(C)** and alternative **(D)** Munc13-4 isoform expression using isoform-specific antibodies in platelets and neutrophils and monocytes, B cells, naïve and memory CD4^+^ T cells, naïve, memory and effector CD8^+^ T cells and NK cells freshly isolated and sorted from the peripheral blood of healthy donors. The blots shown are representative of five independent donors (different colors represent individual donors). The histograms show expression for the conventional and alternative Munc13-4 expression normalized to actin as fold change relative to B cells. Bars depict mean ± SD, whereas dots represent values for each donor. Data are from five healthy donors. *P*-values were calculated using RM-ANOVA. ns, non-significant, *P ≤ 0.05, **P ≤ 0.01; ***P ≤ 0.001; *N* = 3.

Next, we determined if both Munc13-4 isoforms were expressed at the protein level. The conventional and alternative isoforms have a predicted length of 1,090 and 1,071 amino acids, respectively. As the Munc13-4 isoforms only differ in length by 19 amino acids, they have very similar predicted molecular weights. Thus, previously available antibodies to shared epitopes cannot be used to effectively discriminate the isoforms by Western blotting. To demonstrate expression of the alternative Munc13-4 protein and examine the relative expression patterns of either isoform, monoclonal rabbit antibodies specific for the unique Munc13-4 isoform N-termini were tested (Figure 2A). Specificity of the two antibodies was confirmed by transfection of HEK-293T cells with plasmids encoding the conventional or alternative Munc13-4 isoforms, respectively, followed by Western blotting of whole cell lysates (Figure 2B). Next, hematopoietic cell subsets were sorted by flow cytometry (Figure S2), and expression of the two distinct Munc13-4 isoforms were examined in different human peripheral blood cells (Figures 2C,D). Whole cell lysates were blotted with the Munc13-4 isoform-specific antibodies, with actin serving as loading control. Of note, the freshly isolated, unstimulated lymphocyte subsets were of distinct differentiation status yet displayed a similar cellular size. In terms of conventional Munc13-4 (Figure 2C), B cells, naïve and memory CD4^+^ as well as naïve CD8^+^ T cells subsets displayed low levels of expression, with higher expression in neutrophils, platelets, monocytes and differentiated CD8^+^ T cells and NK cells relative to B cells. The expression of conventional Munc13-4 was highest in monocytes as compared to all other subsets. Neutrophils displayed higher levels of conventional Munc13-4 isoform expression as compared to platelets. Notably, compared to naïve CD8^+^ T cells, cytotoxic effector memory CD8^+^ T_EMRA_ cells as well as NK cells expressed significantly higher levels of the conventional Munc13-4 isoform, indicating that the expression of the conventional Munc13-4 isoform is upregulated upon cytotoxic lymphocyte differentiation. In contrast, the alternative Munc13-4 isoform displayed low expression in B cells and monocytes, with significantly higher expression in lymphocyte subsets and platelets as compared to monocytes (Figure 2D). Moreover, alternative Munc13-4 was more abundantly expressed in naïve and memory CD8^+^ T cells as well as NK cells as compared to neutrophils. Contrasting with the conventional isoform, the alternative Munc13-4 isoform was not increased upon lymphocyte differentiation. In summary, our data establish expression of the alternative Munc13-4 isoform in lymphocytes and platelets and reveal that the conventional Munc13-4 isoform represents the predominant isoform in non-lymphoid cells.

### Munc13-4 Isoforms With Distinct N-Termini Display a Similar Subcellular Localization

**Figure 3:**
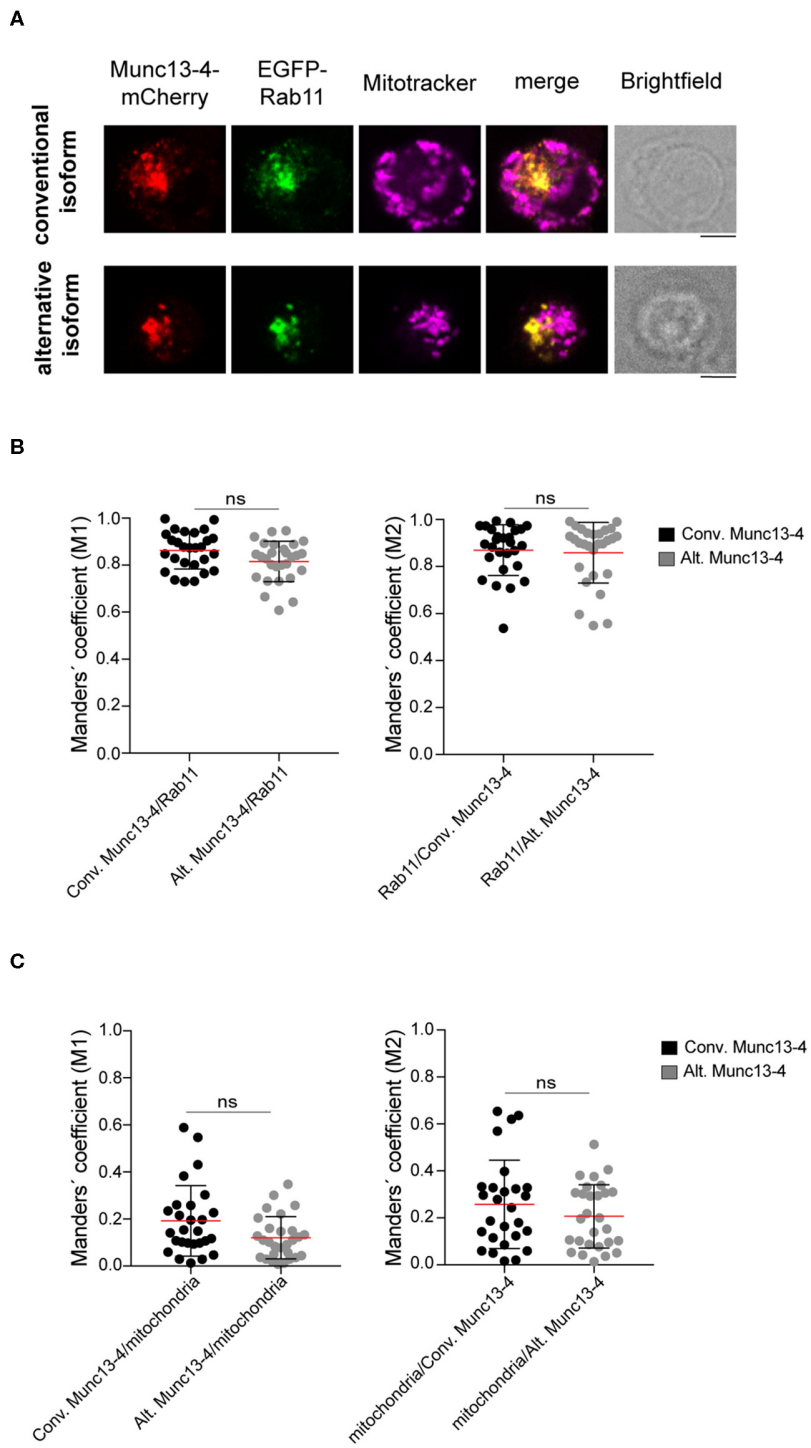
Colocalization of Munc13-4 isoforms with intracellular vesicular compartments. **(A)** Cytotoxic CD8^+^ T cells from healthy donors were transfected with EGFP-Rab11a and conventional (top) or alternative (bottom) Munc13-4-mCherry, stained with Mitotracker to label mitochondria and imaged by confocal microscopy. Representative images are shown. **(B,C)** Quantification of Munc13-4 and Rab11 **(B)** or Munc13-4 and mitochondria **(C)** colocalization was calculated as Manders' overlap coefficient. Plots represent cumulative data from two healthy donors (*n* = 27 cells for conventional Munc13-4, *n* = 28 cells for alternative Munc13-4). Data from each donor are displayed individually in Figure S4. Bars depict mean ± SD, whereas dots represent single cells. *P*-values were calculated using a two-sided Mann-Whitney test. ns, non-significant. Scale bar indicates 5 μM.

Munc13-4 interacts with CG-associated Rab27 through a binding site on Munc13-4 located between amino acids 240–402 (24–26). Since this region is shared between the conventional and alternative Munc13-4 isoforms, both are expected to interact with Rab27a. In addition to Rab27a, Munc13-4 associates with Rab11^+^ recycling endosomes in cytotoxic lymphocytes (27) and neutrophils (28). In cytotoxic T cells, Munc13-4 localizes to and promotes the coalescence of Rab11^+^ endosomes with Rab7^+^/Rab27a^+^ endosomes to generate a precursor to CGs. Deletion of the Munc13-4 C-terminus significantly reduced association with Rab11 (28). Only full-length Munc13-4 is able to induce intracellular compartment coalescence (27), indicating that other Munc13-4 domains contribute to fusion of Rab11^+^ endosomes with Rab7^+^/Rab27a^+^ endosomes. Based on this observation and knowledge of neuronal Munc13 protein localization being controlled by N-terminal interactions, we speculated that the distinct Munc13-4 N-termini could differentially dictate localization of the two isoforms. Thus, we investigated the intracellular distribution of the two Munc13-4 isoforms relative to Rab11^+^ endosomes as well as mitochondria, which we included as negative control. Freshly isolated CD8^+^ T cells from healthy donors were co-transfected with constructs expressing recombinant Munc13-4 isoforms terminally tagged with mCherry as well as EGFP-Rab11 to track the recycling endosomes. Cells were stained with mitotracker to label mitochondria prior to fixation and then imaged by confocal microscopy. Western blot analyses indicated that on average the conventional Munc13-4-mCherry was expressed 16-fold higher than total endogenous Munc13-4, whereas alternative Munc13-4-mCherry was expressed 23-fold higher than total endogenous Munc13-4 (Figures S3A,C). Comparing relative expression of specific isoforms, conventional Munc13-4-mCherry was expressed 22-fold higher than endogenous conventional Munc13-4, whereas alternative Munc13-4-mCherry was expressed 17-fold higher than endogenous alternative Munc13-4 (Figures S3B,D). Although these comparisons using different antibodies did not facilitate an exact determination of the proportion of the two isoforms, data suggest similar expression levels of either Munc13-4 isoform. Confocal imaging revealed that both isoforms showed a similar localization pattern with Munc13-4 mCherry signal predominantly overlapping with EGFP-Rab11^+^ recycling endosomes, but not colocalized with mitochondria (Figure 3A). Co-localization analysis calculating Manders' coefficient confirmed that both conventional and alternative Munc13-4 have a high degree of localization with Rab11^+^ endosomes (Figure 3B), while none of the two isoforms localized with mitochondria in CTLs (Figure 3C). Together, these data showed that both isoforms have a similar intracellular distribution in CTLs, associating with recycling endosomes.

### Both Munc13-4 Isoforms Are Recruited to Cytotoxic Granules and to the Immune Synapse Upon Stimulation

**Figure 4:**
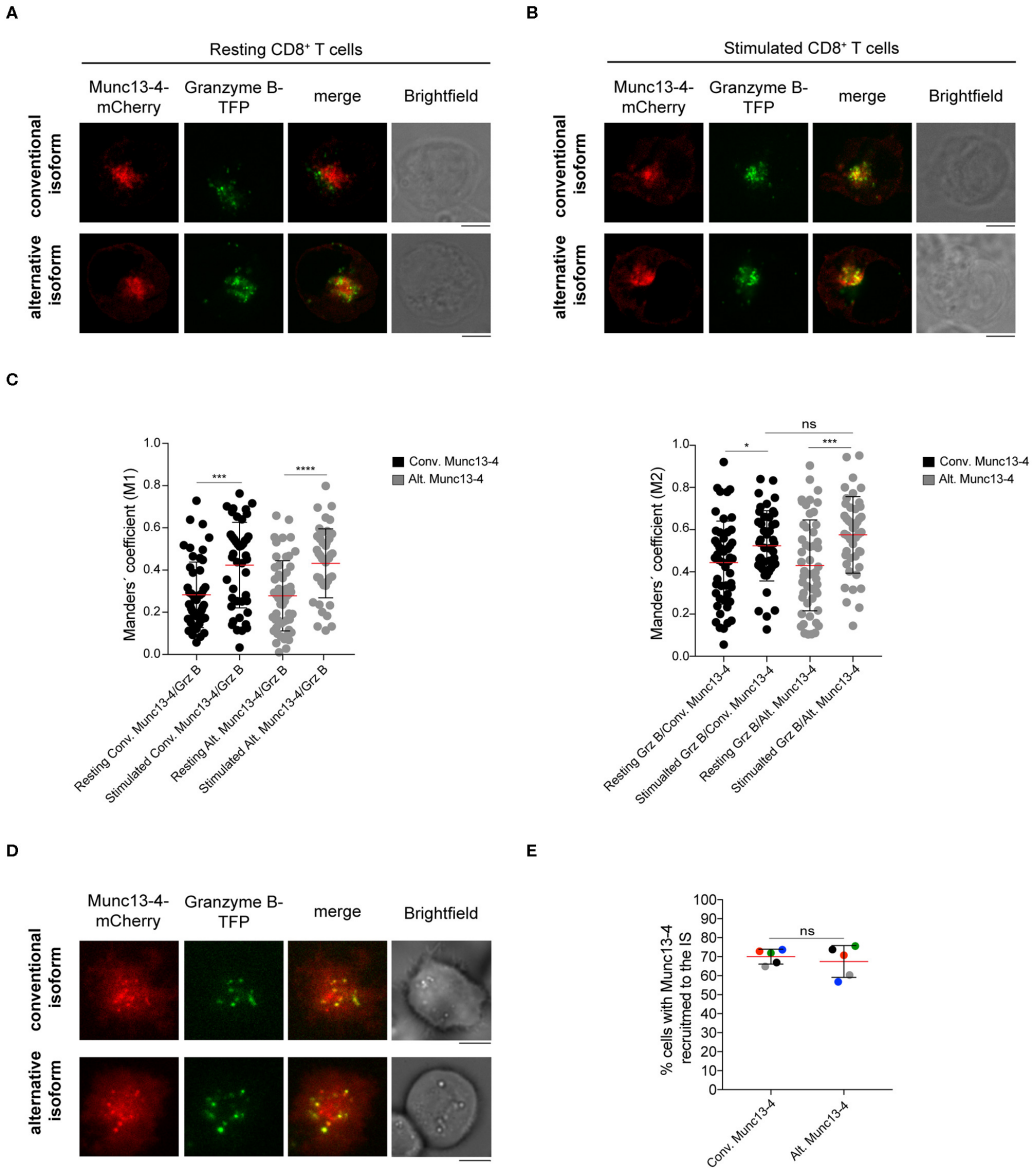
Recruitment of both Munc13-4 isoforms to cytotoxic granules and to the immune synapse. **(A,B)** Confocal microscopy images of cytotoxic CD8^+^ T cells from healthy donors transfected with plasmids encoding Granzyme B and conventional or alternative Munc13-4-mCherry. Images show representative cells **(A)** resting or **(B)** stimulated with PMA plus ionomycin for 20 min at 37°C prior fixation. Upper panels show cells transfected with conventional Munc13-4-mCherry, whereas lower panels show cells transfected with alternative Munc13-4-mCherry. **(C)** Colocalization of Munc13-4 isoforms and Granzyme B in both resting and stimulated conditions was calculated as Manders' overlap coefficient. Plots represent cumulative data from three healthy donors (*n* = 51 cells for conventional Munc13-4, *n* = 52 cells for alternative Munc13-4 from resting CD8^+^ T cells; *n* = 44 cells for both Munc13-4 isoform from stimulated CD8^+^ T cells). Bars depict mean ± SD, whereas dots represent single cells. *P*-values were calculated using a two-sided Mann-Whitney test. ns, non-significant *P ≤ 0.05, ***P ≤ 0.001; ***P ≤ 0.0001. **(D)** TIRF microscopy of cytotoxic CD8^+^ T cells from healthy donors transfected with Granzyme B-TFP and conventional (top) or alternative (bottom) Munc13-4-mCherry. Images show the recruitment of specific Munc13-4 isoforms together with cytotoxic granules to the interface with anti-CD3 mAbs coated coverslips. **(E)** The histogram shows the quantification (%) of cells harboring Munc13-4 in the TIRF plane. Accumulated data from five healthy donors are depicted (different colors represent individual donors). An average of 32 cells were analyzed for each donor. Bars depict mean ± SD. *P*-values were calculated using a paired *T*-test. ns, non-significant. Scale bars indicate 5 μm.

In T cells and NK cells, Munc13-4 isoform is recruited to CG upon stimulation (27, 29). To elucidate whether the distinct N-terminal domains could differentially affect the association of the two isoforms with CGs, we compared the subcellular localization of the conventional and alternative Munc13-4 isoforms in both resting and stimulated CTLs. Freshly isolated CD8^+^ T cells from healthy donors were co-transfected with constructs encoding recombinant conventional or alternative Munc13-4-mCherry and Granzyme B-TFP, as a marker for CGs. Cells were incubated with media alone or with PMA ionomycin and then fixed and imaged by confocal microscopy. We found that both isoforms had a subcellular distribution which did not overlap with that of CG in unstimulated cells (Figure 4A). In contrast, we observed a considerable overlap between both conventional and alternative Munc13-4 mCherry signals with Granzyme B-TFP in stimulated cells (Figure 4B). Colocalization analysis calculating Manders' coefficient showed a significant increase in the degree of co-localization between both Munc13-4 isoforms with CGs in stimulated cells compare to resting conditions (Figure 4C). Furthermore, no difference emerged between conventional and alternative Munc13-4 in terms of associating with CGs in both unstimulated and stimulated cells. Altogether, these data indicate that both conventional and alternative Munc13-4 are similarly recruited to CGs upon stimulation, confirming a similar intracellular distribution of the two isoforms.

We next analyzed Munc13-4 isoform trafficking and distribution to the IS. To do this, we took advantage of high-resolution total internal reflection fluorescence (TIRF) microscopy that generates an evanescent wave within ~200 nm of the plasma membrane allowing a better visualization of vesicle dynamics in the proximity of the IS (18, 19, 30, 31). Freshly isolated CTLs were co-transfected with constructs encoding recombinant Munc13-4-mCherry as well as Granzyme B-TFP and then were placed on coverslips coated with anti-CD3 antibodies to mimic IS formation. Cells were then imaged by live-TIRF microscopy for 10 min. We observed that both isoforms localized at the IS where CGs also accumulated before fusion with the plasma membrane (Figure 4D). No difference in the percentage of cells harboring Munc13-4 signal at the IS was observed between the two isoforms, indicating that both conventional and alternative isoform are similarly recruited to the IS (Figure 4E). Altogether, these data suggest that the N-termini of distinct Munc13-4 isoforms do not impact the activation-induced recruitment of the two isoforms to the CGs as well as their trafficking to the IS. Moreover, since both conventional and alternative Munc13-4 isoforms are recruited to the IS, they can potentially participate in priming and fusion of CGs with the plasma membrane.

### Both Munc13-4 Isoforms Similarly Rescue Exocytosis in CD8^+^ T Cells From FHL3 Patients

**Figure 5:**
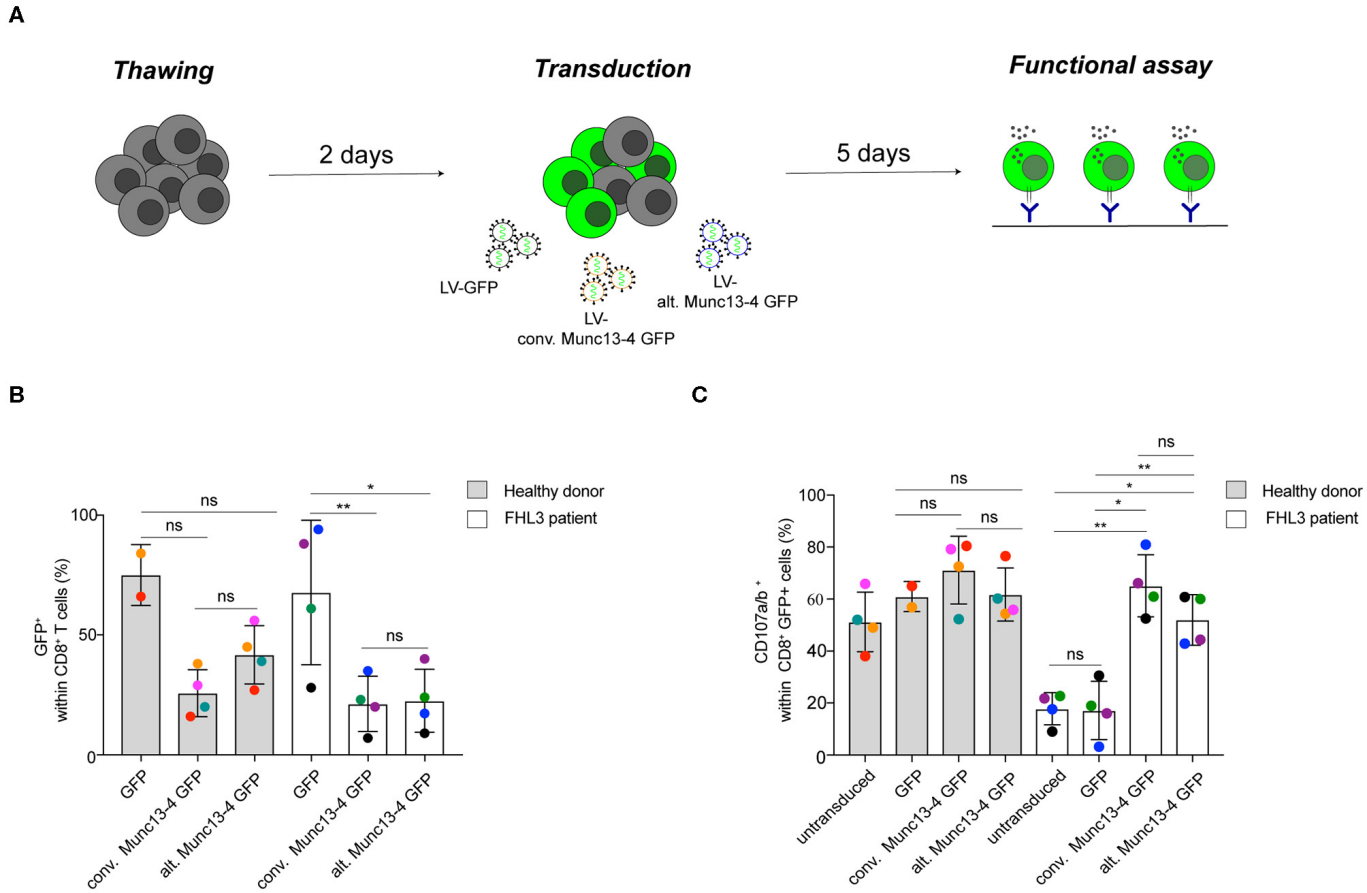
Restoration of T cell exocytosis following conventional or alternative Munc13-4 gene transfer into Munc13-4–deficient T cells. **(A)** Experimental workflow of Munc13-4 isoform restoration experiment transducing PBMCs from FHL3 patients. **(B)** The histogram shows the percentage of GFP^+^ cells 5 days after transduction. **(C)** Flow cytometry analysis of degranulation of cytotoxic CD8^+^ T cells from healthy donors and FHL3 patient cells. Surface expression of CD107a/b was determined by flow cytometry after 3 h stimulation with murine anti-CD3 (30 μg/mL). The histograms show the number of CD107a/b^+^ cells within CD8^+^ T cell population. In transduced samples, the percentage of CD107a/b^+^ cells are presented with respect to GFP^+^ or Munc13-4/GFP^+^ gated cells. Data are from four healthy donors and 4 FHL3 patients. *P*-values were calculated using paired *T*-test for CD107a/b surface expression in the degranulation assay. ns, non-significant *P* > 0.05; *P ≤ 0.05, **P ≤ 0.01. *N* = 2. H.D., healthy donors.

It has previously been shown that transduction of the conventional Munc13-4 isoform can restore exocytosis of Munc13-4-deficient T cells from FHL3 patients (9). As such, constructs encoding the conventional Munc13-4 isoform are being optimized for gene therapy of FHL3 (17). To determine whether the alternative Munc13-4 isoform can support CG exocytosis and compare its activity to that of the conventional Munc13-4 isoform, we performed a genetic rescue experiment of both isoforms in Munc13-4-deficient CD8^+^ T cells from selected FHL3 patients with biallelic *UNC13D* nonsense mutations (Table S1). Peripheral blood mononuclear cells were isolated from healthy volunteers or FHL3 patients, stimulated for 2 days with T cell agonists, and transduced at a multiplicity of infection (MOI) of 100 with either a VSVG-lentiviral vector (LV) encoding GFP or human Munc13-4 isoforms C-terminally tagged with GFP (Figure 5A). An evaluation of the transduction efficiency in transduced CD8^+^ T cells revealed that the VSVG LV system efficiently expressed both Munc13-4 isoforms at comparable levels in CD8^+^ T cells from healthy donors as well less permissive FHL3 patients (Figure 5B). We next quantified the capacity of the two Munc13-4 isoforms to restore exocytosis of FHL3 patient CD8^+^ T cells using a flow cytometry-based assay. Transduced and untransduced cells from healthy volunteers and FHL3 patients were stimulated with anti-CD3 antibodies, stained with antibodies to lineage markers as well as CD107a and CD107b. CD107a and CD107b are contained within late endosomes and lysosomes of cytotoxic lymphocytes and become exposed on the plasma membrane upon cell stimulation, serving as a marker of CG exocytosis. Compared to untransduced or GFP-transduced cells, transduction of both conventional and alternative Munc13-4 isoform restored CG exocytosis in CD8^+^ T cells from the FHL3 patients (Figure 5C). Notably, no significant difference in the ability to restore exocytosis was observed between the two Munc13-4 isoforms. Moreover, overexpression of either Munc13-4 isoform in cells from healthy volunteers did not significantly increase exocytosis as compared to GFP only transduced cells. Collectively, both Munc13-4 isoforms could restore exocytosis in Munc13-4-deficient CD8^+^ T cells.

## Discussion

A large number of genes use different TSSs, with intronic promoters potentially driving the expression of alternative transcripts that lack upstream exons and thereby result in unique N-terminal protein sequences. The expression of distinct isoforms can be intricately regulated and dysregulation of alternative intronic promoters has been associated with developmental defects and diseases (20). Here, we demonstrate that the alternative promoter in *UNC13D* intron 1 controls expression of a functional Munc13-4 isoform which can mediate CG exocytosis in cytotoxic lymphocytes. Contrary to our initial hypothesis, our data indicate that the conventional and alternative Munc13-4 isoforms similarly rescue exocytosis by FHL3 patient CD8^+^ T cells, indicating that the distinct N-termini of these Munc13-4 do not differentially modulate the exocytic pathway.

Several examples of how distinct N-termini can differentially regulate protein function have been described. For example TCF7L2, a member of the transcription factor family TCF/LEF (T-cell transcription factor/lymphoid enhancer factor), encodes a canonical nuclear β-catenin domain-containing isoform that activates target gene transcription in addition to a shorter alternative isoform with an antagonistic function (32, 33). In terms of Munc13-mediated exocytosis, the *Unc13* gene in *C. elegans* encodes for two distinct neuronally expressed isoforms, a longer isoform denoted UNC-13L-R is localized in presynapses and required for normal neurotransmitter release, whereas a short UNC-13M-R isoform is more widely distributed throughout neurons with an apparent minimal role in synaptic transmission (34, 35). The expression of the shorter isoforms is driven by an alternative promoter within *Unc13* intron 13 (34). In mammals, *UNC13B* encodes for two distinct Munc13-2 isoforms, a longer isoform which is ubiquitously expressed and contains an N-terminal C2A domain, while the other isoform is brain-specific and has a shorter N-terminus that lacks the C2A domain (6, 36). The N-terminal C2A domain, which is also present in Munc13-1, is a hub for interactions with active zone proteins including Piccolo, Bassoon, CAST and Rim (37). We hypothesized that the distinct N-termini of conventional and alternative Munc13-4 isoforms might endow hematopoietic cell types with different Munc13-4 mediated capacities for vesicle fusion and exocytosis. Our data from cytotoxic lymphocytes however do not support this hypothesis. The similar function of the two Munc13-4 isoforms might be explained by the fact that the short but distinct amino acid sequence of their N-termini are not associated with a specific function required for exocytosis. Compared to exons encoding for Munc13-4 functional domains, e.g., the C2B, MHD1, MHD2 and C2C domains, the exons encoding for the N-termini of conventional and alternative isoforms display a lower evolutionary sequence conservation through vertebrate evolution (Figure S5). As such, they might not encode specific, evolutionary conserved functions. Notably, multiple alignments of these two exons using only mammalian species showed that they are fairly conserved within mammals (data not shown). This might indicate that the distinct TSS have been conserved through mammalian evolution to regulate Munc13-4 protein levels or stability in different cell types.

Munc13-4 is required for exocytosis by various immune cells, including cytotoxic lymphocytes where hypomorphic mutations result in a reduced frequency and intensity of CG exocytosis (9, 38). Munc13-4 also regulates dense core granule secretion by platelets, with defective platelet exocytosis in *Jinx* mice that have an *Unc13d* splice site mutation (39, 40). Defective exocytosis by *Jinx* mouse platelets was rescued in a dose-dependent manner by reintroduction of Munc13-4 and was also augmented in human platelets upon increasing concentrations of Munc13-4 reconstitution (24, 39). These observations demonstrate that Munc13-4 is a rate-limiting protein for lymphocyte and platelet exocytosis. Furthermore, Munc13-4 is critical for granule and Rab11^+^ vesicle release by neutrophils (28, 41). Although functionally equivalent, we demonstrate that the two Munc13-4 isoforms are differentially expressed in hematopoietic subsets. The conventional Munc13-4 is more highly expressed in myeloid cells as compared to lymphoid subsets, while the alternative Munc13-4 isoform is preferentially expressed in lymphocytes and platelets. These differences were supported by transcriptional data. Cell-type specific transcription factor complexes can promote the expression of conventional vs. alternative isoform in distinct hematopoietic subsets. Moreover, we also found that expression of conventional Munc13-4 is increased in more differentiated CD8^+^ T_EMRA_ and NK cell compared to naïve CD8^+^ T cells, while the alternative isoform is expressed at a comparable level in these subsets. In agreement with these findings, we have previously shown that Munc13-4 expression is up-regulated approximately 5-fold during lymphocyte differentiation (14). Our data therefore suggest that while transcript and protein expression levels of either Munc13-4 isoform are similar in T cells, it primarily is the conventional isoform that is induced upon lymphocyte differentiation, implicating a differential transcriptional regulation of the two isoforms.

With regard to cell function, we speculate that expression of the alternative Munc13-4 may boost the overall exocytic capacity of lymphocytes upon differentiation and in platelets, as the two different isoforms may additively increase exocytosis. Notably, in stimulated T cells from healthy individuals, transfection of either isoform did not augment exocytosis. It is possible that in such stimulated T cells, as well as differentiated cytotoxic lymphocytes, expressing high levels of Munc13-4, other proteins are limiting with respect to the exocytic capacity. According to our data, neutrophils appear less dependent on the expression of the alternative isoforms to regulate granule exocytosis. It is therefore of interest to examine neutrophil and platelet exocytosis in patients with the *UNC13D* intronic mutation, because patients have defective lymphocyte exocytosis but may retain neutrophil and platelet exocytosis. Although these cellular differences in Munc13-4 isoform expression could thus have some clinical impact e.g., with respect to susceptibility to bacterial infections, patients with the *UNC13D* intronic mutation have nonetheless been reported to present with early onset HLH. Munc13-4 has recently been implicated in the regulation of vesicular trafficking pathways in other cell types (23, 41), including granule exocytosis in mast cells (42–44), exosome release by breast cancer cells (45), and secretory granules by endothelial cells (46). Having only tested the function of two Munc13-4 isoforms in the setting of cytotoxic lymphocyte exocytosis, we cannot rule out a different contribution of the two isoforms to vesicle fusion in other cell types. Notably, we find negligible expression of Munc13-4 in transcriptional data from non-hematopoietic cells.

In summary, our study demonstrates that the *UNC13D* intronic promoter encodes a functional Munc13-4 that was able to restore defective CG exocytosis in T cells from FHL3 patients. Nowadays, hematopoietic stem cell transplantation represents the only cure for patients with FHL, but suitable donors cannot always be identified. Gene therapy is therefore being optimized based on lentiviral transduction of hematopoietic stem cells (17, 47). Our work confirmed that the lentiviral system is a successful *in vitro* strategy to correct defective hematopoietic exocytosis in patient cells. Furthermore, having demonstrated that both *UNC13D* isoforms equally contribute to CG degranulation, either isoform could potentially be used to design constructs for gene therapy.

## Data Availability Statement

All datasets generated for this study are included in the article/Supplementary Material.

## Ethics Statement

The studies involving human participants were reviewed and approved by Regional Ethics Review Board in Stockholm (2006/229-31/3; 2013/1273-31/4) and Ethics Commission of the Saarland University Medical Center (2015/83/15; 2015/84/15). Written informed consent to participate in this study was provided by the participants' legal guardian/next of kin.

## Author Contributions

DG, TS, MV, FC, IA, JR, MC, and YB conceived and designed the study. DG, TS, MV, and LT-R performed experiments. DG and TS analyzed data. BT performed bioinformatics analysis. DG and YB wrote the manuscript. All authors contributed to manuscript editing, read and approved the submitted version.

## Conflict of Interest

The authors declare that the research was conducted in the absence of any commercial or financial relationships that could be construed as a potential conflict of interest.
