# Supplementary material for: Alternative UNC13D Promoter Encodes a Functional Munc13-4 Isoform Predominantly Expressed in Lymphocytes and Platelets
Source: Front Immunol. 2020 Jun 9;11:1154. doi: 10.3389/fimmu.2020.01154 (PMC7296141; doi:10.3389/fimmu.2020.01154)
Supplement: Supplementary file 1 [file Data_Sheet_1.docx]

Supplementary Material

## Supplementary Figures

**Figure S1**. (**A**). FANTOM5 CAGE data in primary cells or hematopoietic derived cell line. The histograms showed Tags per million at the different *UNC13D* TSS from the FANTOM5 CAGE data. *TSS*, Transcription Start Site; *CAGE*, Cap Analysis Gene Expression.

**Figure S2. (A) Sequential sorting gating strategy to isolate distinct hematopoietic subsets.** PBMCs from healthy volunteers were gated on forward scatter (FSC) versus side scatter (SSC) plot. Single, live cells were gated on an FSC/SSC plot and antibodies that specifically recognize the cell-surface markers were used to identify NK cells, B cells, naïve CD4^+^ T cells, memory CD4^+^ T cells, naïve CD8^+^ T cells, memory CD8^+^ T cells, effector memory CD8^+^ T_EMRA_ and monocytes.


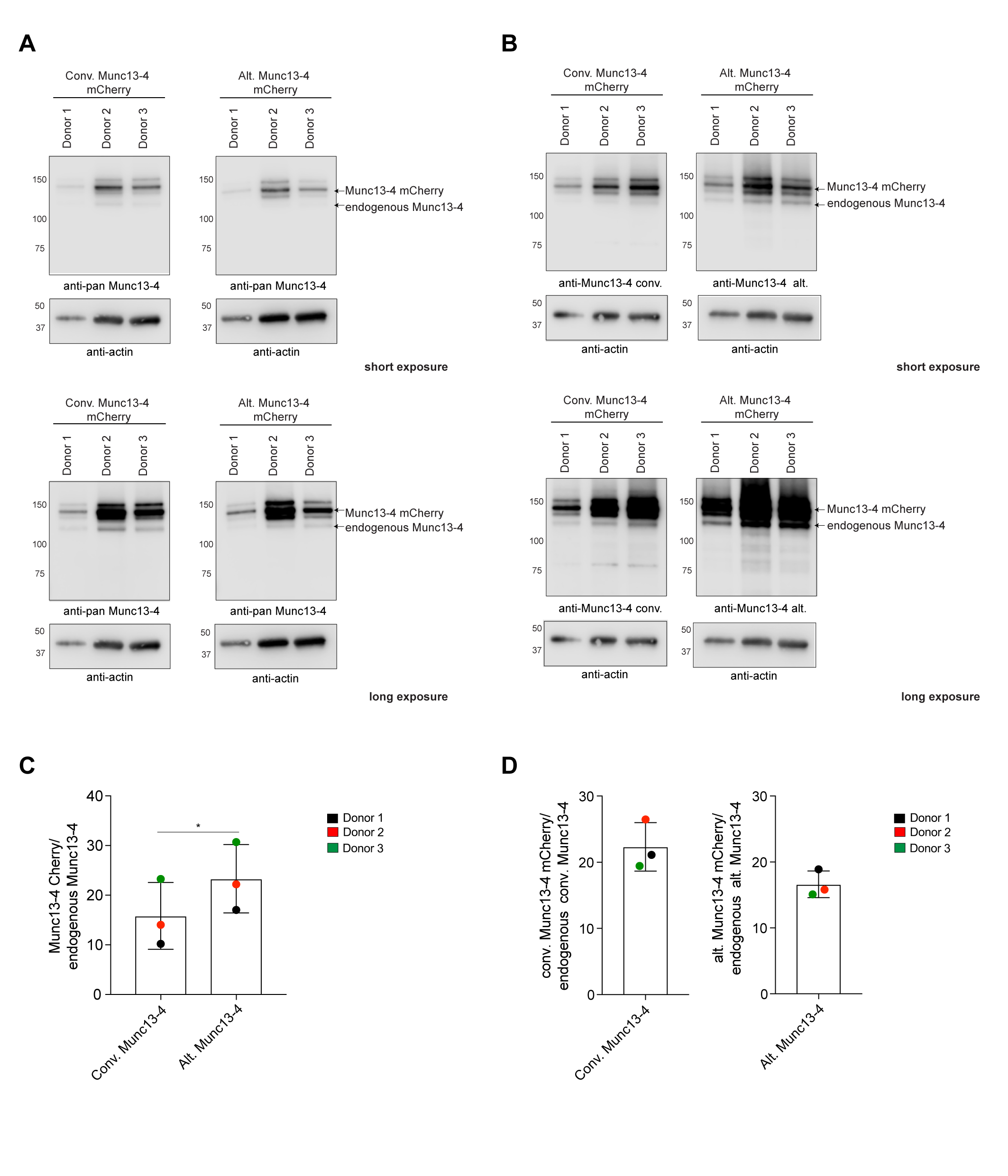


**Figure S3. Evaluation of recombinant Munc13-4-mCherry overexpression.** (**A, B)** Western blot analysis of whole cell lysates from cytotoxic CD8^+^ T cells form healthy donors transfected with Munc13-4-mCherry analyzed in the imaging experiments. Munc13-4 isoform over-expression was detected using (**A**) an anti-pan-Munc13-4 or (**B**) antibodies specific to either isoform. Blotting for actin served as a loading control. (**C, D)** The histograms depict the relative expression of recombinant Munc13-4 mCherry to the total pool of Munc13-4 detected with (**C**) pan antibody or (**D**) endogenous conventional or alternative Munc13-4. The blots shown are representative of 3 independent donors (different colors represent individual donors). Bars depict mean±SD, whereas dots represent values for each donor. P values were calculated using paired T test; *P ≤ 0.05.


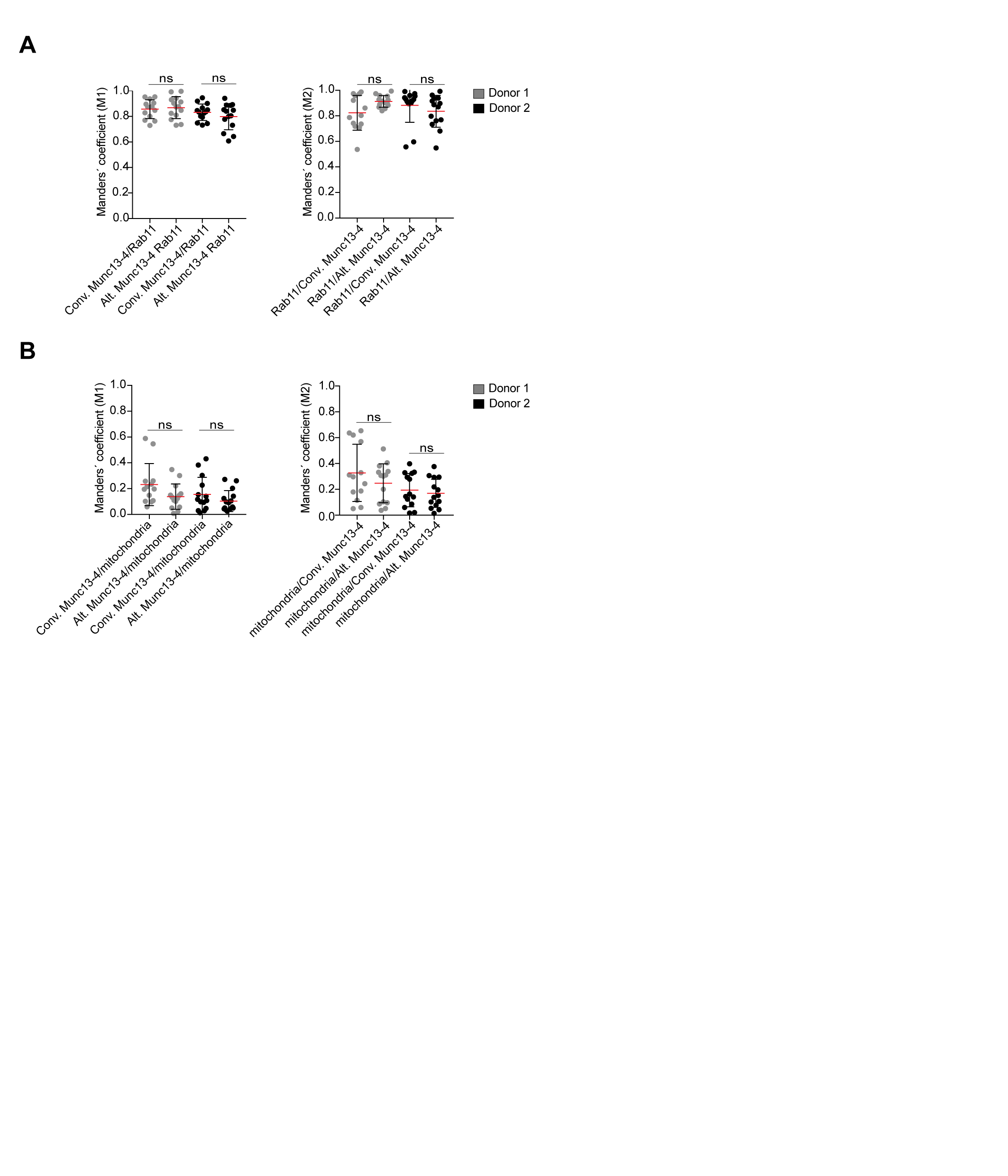


**Figure S4.** **Quantification of Munc13-4 isoform colocalization with Rab11 or mitochondria in individual donors**. (**A,** **B)** Colocalization between (A) Munc13-4 and Rab11 or (B) Munc13-4 and mitochondria was calculated as Manders’ overlap coefficient for each of the two donors analyzed in Figure 3. Plots represent data from 13 cells for conventional Munc13-4 and 14 cells alternative Munc13-4 from Donor 1 and from 14 cells for both Munc13-4 isoforms from Donor 2. Bars depict mean±SD for each donor, whereas dots represent single cells. P values were calculated using a two-sided Mann-Whitney test. ns, non-significant.

**Figure S5. Conventional and alternative N-terminal exons are less conserved than exons encoding Munc13-4 functional domains. (A)**. Schematic representation of human *UNC13D* gene mapping the exons corresponding to the functional Munc13-4 protein domains and the distinct N-terminal of the alternative and conventional isoform. (**B)**. Evolutionary conservation profile of Munc13-4 regions highlighted in A. The evolutionary nucleotide conservation score was calculated through “Phast Cons tool” from UCSC Genome browser.

Table S1

Features of Enrolled FHL3 patients

|  | ***UNC13D* variant** | **Predicted effect  on protein level** | **Exon** | **Ethnicity** | **Gender** | **HLH criteria** |
| --- | --- | --- | --- | --- | --- | --- |
| P1 | Hmz c.762del | p.Cys255Alafs*73 | Exon 24 | Bangladeshi | Female | 5/5 |
| P2 | Hmz c.640C>T | p.Arg214Ter | Exon 8 | Turkish | Male | 6/7 |
| P3 | Hmz c.753+1G>T | Splice error | Exon 9 | Turkish | Male | 5/6 |
| P4 | Hmz c.2346_2349delGGAG | p.Arg782Serfs*12 | Exon 24 | Caucasian/Polish | Male | 5/5 |
